# Expression of truncated human epidermal growth factor receptor 2 on circulating tumor cells of breast cancer patients

**DOI:** 10.1186/s13058-015-0624-x

**Published:** 2015-08-19

**Authors:** Galatea Kallergi, Sofia Agelaki, Maria A. Papadaki, Dimitris Nasias, Alexios Matikas, Dimitris Mavroudis, Vassilis Georgoulias

**Affiliations:** Laboratory of Τumor Cell Biology, School of Medicine, University of Crete, Voutes, 71110 Heraklion, Crete Greece; Department of Medical Oncology, University General Hospital of Heraklion, Voutes, 71110 Heraklion, Crete Greece

## Abstract

**Introduction:**

The truncated form of human epidermal growth factor receptor 2 (p95HER2) lacks the HER2 extracellular domain and has been associated with poor prognosis and resistance to trastuzumab. In the present study, the expression of p95HER2 was investigated on circulating tumor cells (CTCs) from breast cancer patients.

**Methods:**

Triple-staining immunofluorescent experiments were performed on peripheral blood mononuclear cells’ (PBMCs) cytospins obtained from patients with early (*n* = 24) and metastatic (*n* = 37) breast cancer. Cells were stained with the pancytokeratin (A45-B/B3) antibody coupled with antibodies against the extracellular (ECD) and the intracellular (ICD) domains of HER2. Slides were analyzed with either confocal laser scanning microscopy or with the Ariol system.

**Results:**

HER2-positive CTCs were identified in 55.6 % of early and 65.2 % of metastatic CTC-positive breast cancer patients. p95HER2-positive CTCs were identified in 11.1 % of early and 39.1 % of metastatic breast cancer patients (*p* = 0.047). In 14 patients with metastatic HER2-positive breast cancer, CTCs were also analyzed before and after first-line trastuzumab therapy. Trastuzumab reduced the percentage of patients with full-length HER2-positive CTCs from 70 % at baseline to 50 % (*p* = 0.035) after treatment while increased the percentage of patients with p95HER2-positive CTCs from 40 % to 63 %. Moreover, the overall survival of metastatic patients with p95HER2-positive CTCs was significantly decreased (*p* = 0.03).

**Conclusions:**

p95HER2-positive CTCs can be detected in both early and metastatic breast cancer patients. Their incidence is increased in the metastatic setting and their presence is associated with poor survival. Longitudinal studies during anti-HER2 treatment are required to determine the clinical relevance of p95HER2-expressing CTCs.

**Electronic supplementary material:**

The online version of this article (doi:10.1186/s13058-015-0624-x) contains supplementary material, which is available to authorized users.

## Introduction

Human epidermal growth factor receptor 2 (HER2), a member of the epidermal growth factor receptor (EGFR) family, is overexpressed in approximately 20–30 % of human breast cancers, and its expression is associated with poor patient prognosis [1]. It has been suggested that at least part of the prognostic significance of HER2 overexpression may be related to the truncated form of the receptor HER2 (p95HER2), which lacks the extracellular domain (ECD) [2]. Two different mechanisms have been proposed for the generation of p95HER2 receptor: (i) the cleavage by metalloproteinases and (ii) the differential initiation of mRNA translation from an internal AUG codon [3].

In primary breast cancer, p95HER2 expression has been associated with node metastasis [4]. In addition, p95HER2 has been correlated with the extent of lymph node involvement and was found to be increased in metastatic nodes compared to the primary tumor [5]. Furthermore, the expression of p95HER2 but not of the full-length p185HER2 receptor was predictive of reduced 5-year survival in patients with early breast cancer [2].

The humanized monoclonal antibody trastuzumab (Herceptin) was the first HER2-directed agent approved for clinical use in breast cancer patients showing marked clinical benefit for patients with early and metastatic disease [6]. The antibody has single-agent activity against tumor cells and enhances the effectiveness of certain chemotherapeutic agents, mostly taxanes, possibly by inhibiting anti-apoptotic signaling pathways [7, 8]. However, approximately 15 % of patients receiving trastuzumab-based adjuvant chemotherapy will develop metastatic disease. Moreover, many patients with metastatic disease do not respond to trastuzumab therapy or develop refractory disease within 1 year of treatment [9], suggesting the presence of primary or acquired resistance, respectively. In addition, p95HER2 has been associated with resistance to trastuzumab and with sensitivity to lapatinib [10]. Furthermore, it was shown that trastuzumab was ineffective against in vivo tumor growth of T47D breast cancer cells stably transfected with a truncated form of HER2 [11].

Circulating tumor cells (CTCs) have been proposed as a powerful prognostic factor in patients with breast cancer. Moreover, CTCs are considered to be a “real-time liquid biopsy” of the tumor and provide the opportunity of individualizing therapy according to targets present on CTCs rather than the primary tumor [12]. We, among others, have shown that HER2 is expressed on CTCs of early and metastatic breast cancer patients irrespective of the HER2 status of the primary tumor. Specifically, HER2 was expressed more frequently in CTCs than in the corresponding primary tumors [13–15]. Moreover, the presence of HER2-positive CTCs has been associated with poor clinical outcome in early breast cancer [16–18]. Finally, we have recently reported the results of a randomized phase II study showing that “secondary adjuvant” administration of trastuzumab in HER2-negative patients with detectable HER2-positive CTCs after the completion of adjuvant chemotherapy was associated with a significant benefit in disease-free survival (DFS) [19].

To the best of our knowledge, there are no data concerning the expression of p95HER2 on breast cancer patients’ CTCs. In the present study, using a triple immunofluorescence staining, we investigated the presence of p95HER2-positive CTCs in patients with early and metastatic breast cancer as well as the effect of trastuzumab-based chemotherapy on the HER2 status of patients’ CTCs.

## Materials and methods

### Patient samples and cytospin preparation

Sixty-one patients (24 with early and 37 with metastatic breast cancer) were enrolled in this study. Blood samples were obtained before the initiation of adjuvant and first-line treatment, respectively. All blood samples were obtained at the middle of the vein puncture, after the first 5 ml of blood was discarded. These precautions were undertaken in order to avoid contamination of the blood sample with epithelial cells from the skin during sample collection. All patients gave their informed consent to participate in the study, which has been approved by the Ethics and Scientific Committees of University General Hospital of Heraklion, Crete, Greece. Ten female healthy blood donors were also included as negative controls.

Peripheral blood mononuclear cells (PBMCs) were isolated with Ficoll-Hypaque density gradient (d = 1,077 gr/mol) centrifugation at 1800 rpm for 30 min. Peripheral blood mononuclear cells (PBMCs) were washed three times with phosphate-buffered saline (PBS) and centrifuged at 1500 rpm for 10 min. Aliquots of 500,000 cells were cytocentrifuged at 2000 rpm for 2 min on glass slides. Cytospins were dried and stored at −80 °C. Two slides from each patient were used for staining experiments and the results were expressed in CTCs/500,000 examined cells.

### Cell cultures

SKBR3 breast cancer cells (obtained from ATCC; the American Type Culture Collection, Manassas, VA, USA), which overexpress HER2, were cultured in RPMI supplemented with 10 % fetal bovine serum (FBS). Cells were maintained in a humidified atmosphere of 5 % CO_2_ in air. Subcultivation was performed with 0.25 % trypsin and 5 mM EDTA (GIBCO BRL, Life Technologies, Rockville, MD, USA). HER2-positive SCOV3 ovarian cancer cells (obtained from the ATCC) were cultured in McCoy’s medium supplemented with 10 % FBS (GIBCO BRL).

Both cell lines were routinely checked every month for estrogen receptor (ER), progesterone receptor (PR), HER2 and EGFR expression by immunofluorescence staining experiments and reverse transcription polymerase chain reaction (RT-PCR). Cells were maintained in a humidified atmosphere of 5 % CO_2_-95 % air. All experiments were performed during the logarithmic growth phase 15–20 h prior to the experiments. Cells were centrifuged on cytospins according to the procedure followed for patient samples.

### Immunoblot analysis

SKBR3 and SCOV3 cells were lysed using 500 μl cold lysis buffer (50 mM Tris/HCl, 1 % ΤritonX-100 pH 7.4, 1 % sodium deoxycholate, 0.1 % SDS, 0.15 % ΝaCl, 1 mM ΕDTA, 1 mM sodium orthovanadate) at 4 °C and flasks were scraped off. The remaining insoluble material was removed by centrifugation. Protein concentration of the samples (20 μg) was determined using the Bio-Rad protein kit (Bio-Rad Laboratories, Hercules, CA, USA). Equal amounts of protein were subjected to SDS electrophoresis and blotted onto nitrocellulose membrane. Proteins were incubated with antibodies against the: (i) anti-HER2 (H-200) (Santa Cruz Biotechnology, Dallas, TX, USA), against the extracellular domain of the receptor (ii) HER2 (CB11) (Santa Cruz Biotechnology) intracellular domain (ICD); and (iii) anti-actin (Chemicon International, Inc., Temecula, CA, USA), for 1 h at room temperature and, then, with the appropriate secondary antibodies. Detection of protein bands was performed using the ECL kit (Bio-Rad Laboratories). All the proteins were quantified using a PC-based image analysis system (Image Analysis Inc, Toronto, ON, Canada).

### Triple immunofluorescence staining

A triple immunofluorescence staining was developed for the simultaneous detection of cytokeratin (CK) and the extracellular (p95HER2) and intracellular domains of HER2. PBMC cytospins obtained from patients were fixed with cold aceton:methanol 9:1 (vol/vol) for 20 min and stained for HER2 expression using the anti-mouse monoclonal CB11 antibody, which reacts with the ICD of HER2 protein. After incubation with anti-mouse Alexa555 fluorochrome (Molecular Probes, Invitrogen, Carlsbad, CA, USA), cells were incubated with anti-rabbit HER2 (H-200) antibody against an epitope corresponding to amino acids 251–450 mapping within an N-terminal ECD of HER2. Subsequently, cells were incubated with the secondary antibody, Alexa Fluor 633 (Invitrogen) anti-rabbit immunoglobulin G (IgG). Zenon technology (FITC-conjugated IgG1 antibody; Molecular Probes, Invitrogen) was used for CK detection with the A45-B/B3 (Micromet, Munich, Germany) antibody. Zenon antibodies were prepared within 30 min before use. Positive and negative controls were used in each experiment by omitting one primary antibody per slide and incubating the cells with the corresponding IgG isotype bound to the corresponding fluorochrome. All the slides were also stained with DAPI conjugated with antifade reagent (Invitrogen). In addition spiking experiments with 1, 10 and 100 SKBR3 cells in PBMCs from healthy volunteers revealed that our method could identify up to one tumor cell among 10^6^ PBMCs.

Slides were analyzed using confocal laser scanning microscope module (Leica Lasertechnik, Heidelberg, Germany) and/or the automated Ariol microscopy system for CTC identification (Genetix, New Milton, UK). The cytomorphological criteria proposed by Meng et al. [20] (i.e., high nuclear/cytoplasmic ratio, larger cells than white blood cells, etc.) were used in order to characterize a CK-positive cell as a CTC. Samples containing at least one CK-positive cell among 10^6^ PBMCs were considered positive.

HER2-positive CTCs were considered all the CK-positive cells which expressed both the extracellular and the intracellular domain of the receptor while p95HER2-positive CTCs were considered only those which lacked expression of the extracellular domain of HER2. Furthermore, CK-positive/HER2-positive CTCs had to have a HER2 intensity staining higher than the negative control included in each experiment according to standard methodology [14, 15].

### Her2 staining in primary tumors

All the primary tumors were evaluated for HER2 expression using the HercepTest (Dako, Carpinteria, CA, USA) ICH assay. According to the manufacturer recommendations, slides were placed in xylene, absolute ethanol, 95 % ethanol and water sequentially. After epitope retrieval, slides were stained with the corresponding reagent. Slides were then scored according to HercepTest kit guidelines for the interpretation of positivity.

### Statistical analysis

In early breast cancer patients, disease-free interval (DFI), was defined as the time from the initiation of adjuvant treatment until the day of the first evidence of disease recurrence and overall survival (OS) as the time from disease diagnosis to death. In patients with metastatic disease, progression-free survival (PFS) was defined from the initiation of front-line treatment until disease relapse or death. Kaplan-Meier curves and Cox regression analysis for PFS and OS were compared using the log-rank test to provide a univariate assessment of the prognostic value of selected clinical risk factors. Clinicopathologic factors such as menopausal status, tumor size, number of involved lymph nodes, ER and PR status and HER-2 status were also evaluated in univariate analysis. Variables that were found to be significant at the univariate screen were then entered in a stepwise multivariate Cox proportional hazards regression model to identify those with independent prognostic information. All statistical tests were performed at the 5 % level of significance. SPSS version 15 (SPSS Inc, Chicago, IL, USA) statistical software was used for the analysis.

## Results

### Detection of the extracellular and intracellular domain of HER2 in SKBR3 and SKOV3 cell lines

**Fig. 1:**
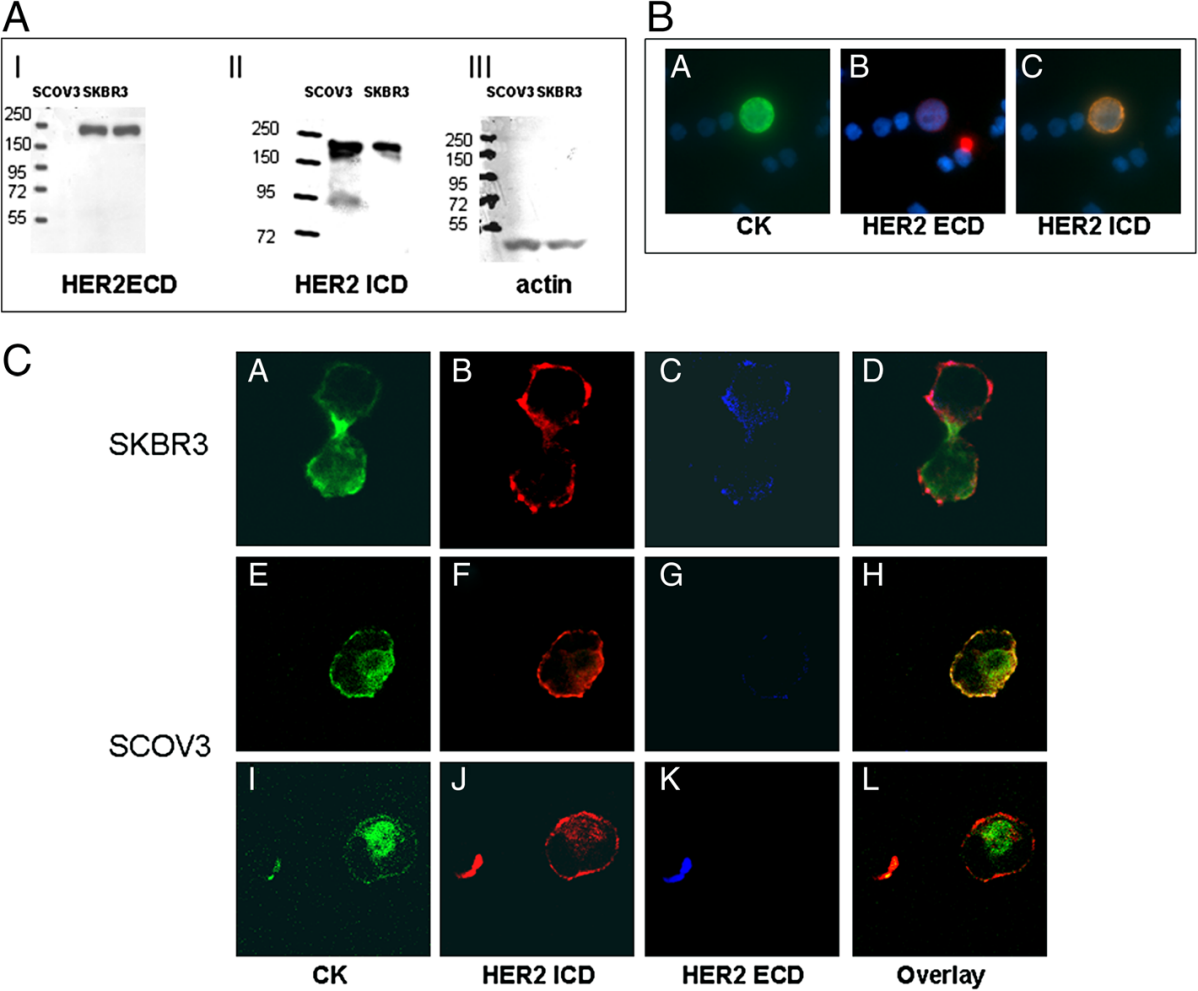
Expression of full-length HER2 and its truncated form on control SKBR3 and SCOV3 cells. **a** Equal protein amounts of SCOV3 and SKBR3 cells were subjected to SDS-PAGE electrophoresis and transferred to nitrocellulose membrane. Total proteins were detected by immunoblotting (IB) with (*I*): anti-HER2 ECD, (*II*) HER2 ICD (CB11 clone) and (*III*) anti-actin. **b** Representative Ariol system images from control slides with SKBR3 cells. (*a*) SKBR3 cells stained with HER2 (ECD)/Alexa633/Alexa555 antibodies (*b*) SKBR3 cells stained with HER2 (ICD)/Alexa633/Alexa555 antibodies and (*c*) SKBR3 cells stained with HER2(ECD)/HER2 (ICD)/Alexa633/Alexa555 HER2 antibodies. **c** Representative confocal laser scanning micrographs (×40) of SKBR3 and SCOV3 cells triple-stained with HER2 (ICD), HER2 (ECD) and pancytokeratin (A45-B/B3) antibodies. ECD and ICD were identified in SKBR3 (*A-D*) cells while low (*E-H*) or no ECD (*I-L*) was identified in SCOV3 cells. *ECD* extracellular domain, *HER2* human epidermal growth factor receptor 2, *ICD* intracellular domain

SKBR3 and SCOV3 cancer cell lines, which express HER2, were used for the evaluation of the triple-staining experiments. SKOV3 cells have been shown to shed lower levels of HER2 extracellular domain (ECD) compared to SKBR3 cells [21] and express both the full-length and the truncated HER2 receptor [4]. Immunoblot analysis, using an antibody against the ECD, showed that both cell lines expressed the full-length receptor [Fig. 1a (I)] whereas blotting with the antibody against the intracellular domain (ICD) revealed that SCOV3 cells express higher levels of an extra fragment of 95 kDa molecular weight [Fig. 1a (II)]. This observation confirmed that both SKBR3 and SCOV3 could be used as controls for full-length and p95HER2 staining experiments.

In order to evaluate the expression of p95HER2 and full-length HER2 receptor in SKBR3 and SCOV3 cells and to verify that these cell lines could be used as controls for staining procedures, triple-staining experiments were performed. Cytospins with SKBR3 and SCOV3 cells were triple-stained with pancytokeratin and antibodies against the extracellular and intracellular domain of HER2 and analyzed both with Ariol system (Fig. 1b) and confocal laser scanning microscopy (Fig. 1c). The results confirmed the presence of SCOV3 cells lacking the ECD (Fig. 1c).

Subsequently, SKOV3 and SKBR3 cell lines were used in each experiment as positive controls for the p95HER2 and the full-length HER2 receptor. Positive [with HER2(ECD)/HER2(ICD)/CK] and negative controls, prepared by omitting each primary antibody and employing only the corresponding IgG isotype, were included in each separate experiment. Control experiments using confocal laser scanning microscopy were also performed to ensure that the bleaching-through phenomenon was avoided.

### Expression of the p95HER2 receptor on CTCs in early and metastatic breast cancer patients

**Fig. 2:**
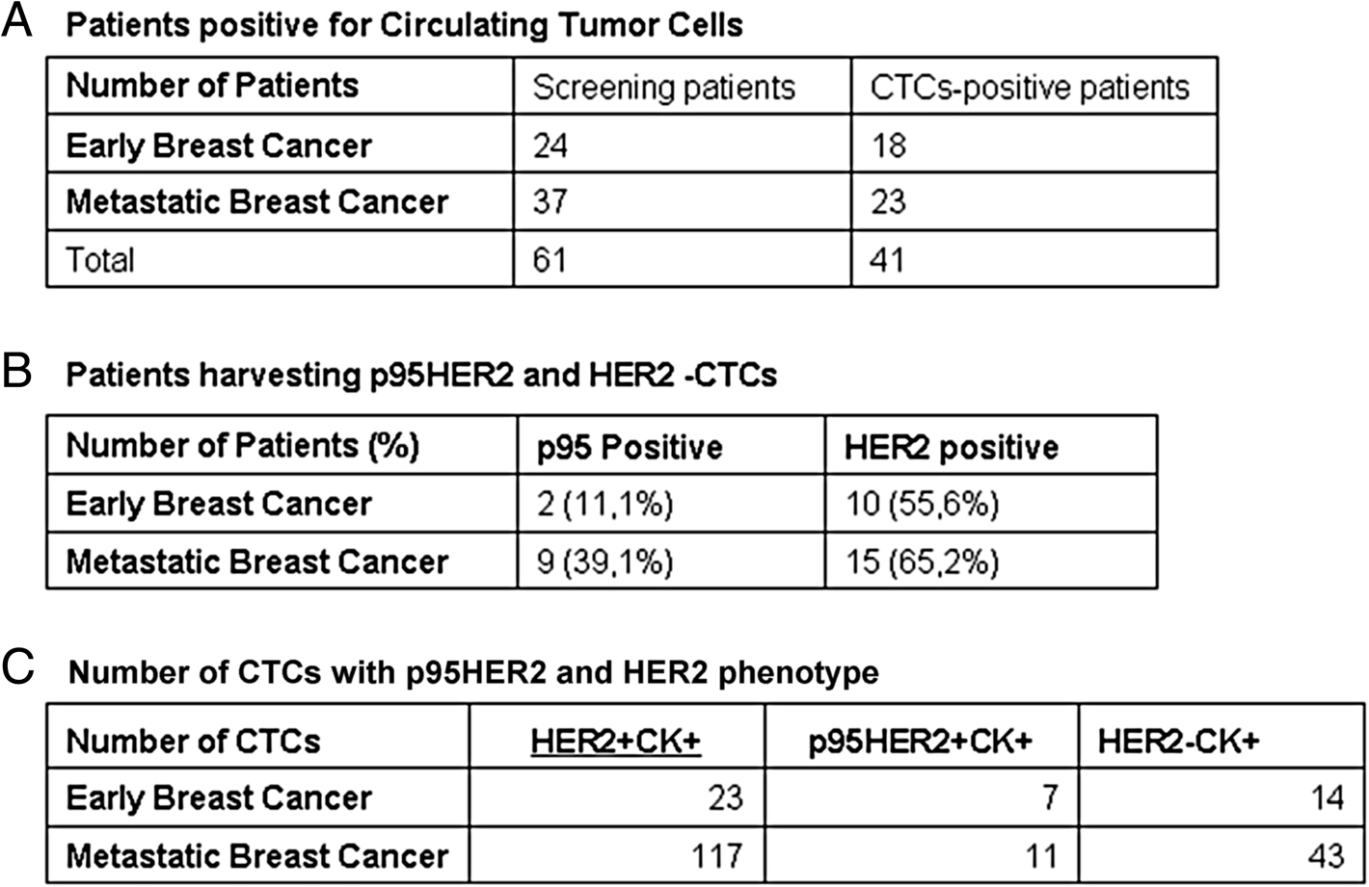
HER2 and truncated HER2 expression in early and metastatic breast cancer patients. **a** Number of patients harvesting CTCs. **b** Number of patients harvesting p95HER2 and HER2 CTCs. **c** Number of CTCs with p95HER2 and HER2 phenotype. *CTCs* circulating tumor cells, *HER2* human epidermal growth factor receptor 2, *p95HER2* the truncated form of HER2

Twenty-four consecutive patients with early and 37 with metastatic breast cancer were included in the study. These patients were found to be CTC-positive when routinely screened for CK-positive/CD45-negative CTCs on PBMC smears. Triple immunofluorescence experiments CK/HER2-(ICD)/HER2-(ECD) have shown that CK-positive cells were observed in 18 early and 23 metastatic patients (Fig. 2a). Patients’ characteristics are shown in Table 1.

**Table 1:** Patients’ characteristics

Early disease (24 patients)		Metastatic disease (37 patients)	
Age	N	Age	N
Median, range	52 (37–72)	Median, range	59 (34–86)
Menopausal status		Menopausal status	
Premenopausal	9 (37.5 %)	Premenopausal	11 (29.7 %)
Postmenopausal	14 (58.3 %)	Postmenopausal	22 (59.5 %)
Unknown	1 (4.2 %)	Unknown	4 (10.8 %)
Tumor size		Disease sites	
pT1	6 (25 %)	1	15 (40.5 %)
pT2	8 (33.3 %)	2	8 (21.6 %)
pT3	1 (4.1 %)	3	5 (13.5 %)
Unknown	9 (37.5 %)	≥4	3 (8.2 %)
		Unknown	6 (16.2 %)
Lymph node status		Predominantly visceral disease	
Node-negative	10 (41.7 %)	Yes	15 (40.5 %)
Node-positive	10 (41.7 %)	No	17 (46 %)
Unknown	4 (16.6 %)	Unknown	5 (13.5 %)
Histologic grade		Primary breast cancer	
Grade 1	0 (0 %)	Adjuvant	13 (35.1 %)
Grade 2	10 (41.7 %)	Metastatic	6 (16.2 %)
Grade 3	9 (37.5 %)	Unknown	18 (48.7 %)
Grade 4	2 (8.3 %)		
Unknown	3 (12.5 %)		
ER/PR tumor status		ER/PR tumor status	
Positive	20 (83.3 %)	Positive	22 (59.5 %)
Negative	1 (4.2 %)	Negative	11 (29.7 %)
Unknown	3 (12.5 %)	Unknown	4 (10.8 %)
HER2 tumor status		HER2 tumor status	
Positive	5 (20.8 %)	Positive	16 (43.2 %)
Negative	17 (70.8 %0	Negative	17 (46 %)
Unknown	2 (8.4 %)	Unknown	4 (10.8 %)

*Abbreviations*: *ER* estrogen receptor, *PR* progesterone receptor, *HER2* human epidermal growth factor receptor 2

HER2-positive CTCs were observed in ten out of 18 (55.6 %) and 15 out of 23 (65.2 %) patients with early and metastatic breast cancer, respectively (Fig. 2b). The p95HER2 receptor was identified in 11.1 % (two out of 18) and in 39.1 % (nine out of 23) patients with early and metastatic disease, respectively (*p* = 0.047). There was no patient with early breast cancer who had CTCs expressing exclusively the p95HER2 form of receptor, whereas in one patient with metastatic disease, only CTCs expressing the p95HER2 receptor could be detected.

Among patients with HER2-positive primary tumors, HER2-positive CTCs could be identified in 50 % and 70 % of patients with early and metastatic disease, respectively. In patients with HER2-negative primary tumors the corresponding numbers were 66.7 % in both settings. In addition, p95HER2-positive CTCs were observed in 0 % and 33.3 % (*p* = 0.229) of early and metastatic patients with HER2-positive primary tumors, respectively. Conversely, in patients with HER2-negative primary tumors, p95HER2-positive CTCs were detected in 16.7 % and 50 % of patients with early and metastatic disease, respectively (*p* = 0.382).

A total of 44 and 171 CTCs were analyzed in patients with early and metastatic breast cancer, respectively. The average percentage of the total examined CTCs per patient that expressed the full-length HER2 receptor was 45 % in early and 34 % in metastatic patients (*p* = 0.388). In addition, 18 % of the total number of examined CTCs expressed the p95HER2 receptor in patients with metastatic disease compared to 6 % in patients with early disease (*p* = 0.059). Furthermore, 49 % and 47 % of the total examined CTCs per patient in early and metastatic disease, respectively, were CK-positive/HER2-negative (Fig. 2c).

**Fig. 3:**
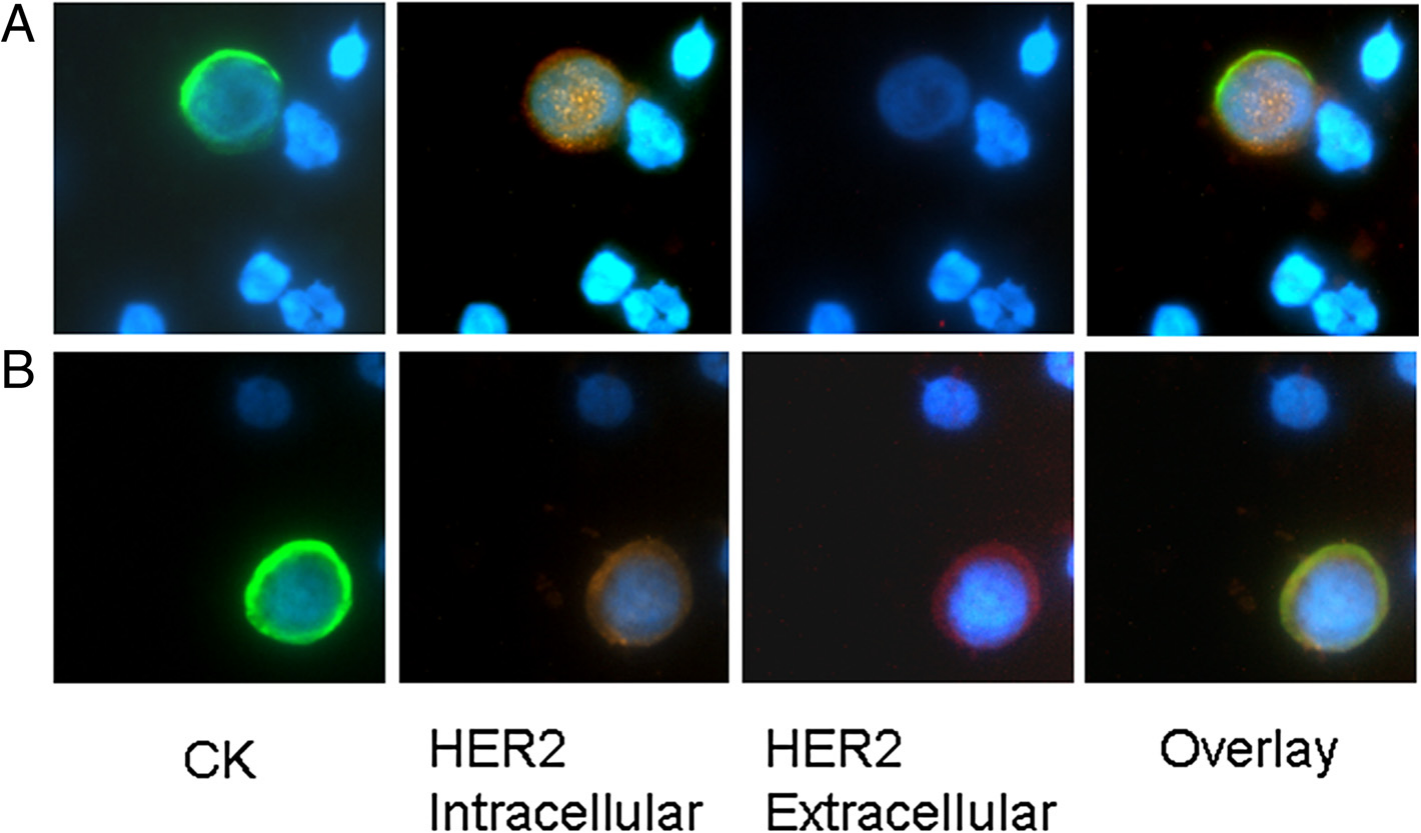
HER2 full-length and truncated HER2 expression on CTCs of breast cancer patients. Representative Ariol system images from one metastatic patient expressing both (**a**) CTCs with truncated HER2 receptor after triple-staining with HER2 (ICD), HER2 (ECD) and pancytokeratin (A45-B/B3) antibodies and (**b**) CTCs with full length receptor. *CTCs* circulating tumor cells, *ECD* extracellular domain, *HER2* human epidermal growth factor receptor 2, *ICD* intracellular domain

Ιn two patients with early (Table 2; patient number 6 and number 13) and in four (Table 2; patient number 7, number 11, number 20 and number 22) with metastatic disease, two distinct subpopulations of CTCs, according to the expression status of HER2, could be identified: CTCs expressing the full-length HER2 receptor as well as CTCs expressing the p95HER2 receptor. Figure 3 demonstrates the intracellular distribution of HER2 in one patient with the two phenotypically distinct subpopulations of CTCs. Particularly, in panel A, the cell lacks the extracellular domain and the distribution of HER2 is mainly cytoplasmic, while in panel B, HER2 extracellular domain is evident along with a mostly membrane localization of its intracellular domain.

**Table 2:** ER/PR/HER2 status of the primary tumor and CTCs in CK-positive breast cancer patients

Early breast cancer patients						
	HER2 primary	ER primary	PR primary	HER2in + HER2ext + CK+	HER2in + HER2ext-CK+	HER2in-HER2ext-CK+
1	+	+	+	0	0	2
2	-	+	+	0	0	1
3	-	+	+	1	0	0
4	+	-	+	0	0	1
5	-	+	+	0	0	1
6	-	+	-	1	3	0
7	-	-	+	1	0	4
8	-			0	0	1
9	-	+	+	1	0	0
10	-	+	+	2	0	0
11	-	+	+	0	0	1
12	-	+	+	2	0	0
13	-	+	+	9	4	0
14	+			2	0	0
15	-			0	0	2
16	-	+	+	3	0	0
17	-	-	+	0	0	1
18	+	+	+	1	0	0
				First-line metastatic breast cancer patients		
	HER2 primary	ER primary	PR primary	HER2in + HER2ext + CK+	HER2in + HER2ext-CK+	HER2in-HER2ext-CK+
1	+			5	0	0
2	-	+	-	0	1	2
3	-	+	+	100	0	0
4	+	-	-	0	0	1
5	-	+	-	0	0	1
6	-	+	+	1	0	0
7	+	-	-	1	1	0
8	-	-	+	0	0	1
9	+	+	+	1	0	0
10	-	+	+	0	0	8
11	-	+	+	2	1	1
12	+	+	+	0	2	1
13	-	+	+	0	0	1
14				0	1	1
15	+	-	-	1	0	0
16				0	0	1
17	+	-	-	1	0	0
18	+	-	-	0	1	0
19	+	-	-	0	0	1
20	-	+	+	2	1	1
21	-	-	-	0	1	1
22		+	+	3	2	20
23	+	+	+	0	0	2

*Abbreviations*: *ER* estrogen receptor, *PR* progesterone receptor, *HER2* human epidermal growth factor receptor 2, *CTCs* circulating tumor cells; *CK* cytokeratin

### HER2 status in primary tumor and CTCs

The HER2 status of the primary tumor is shown in Table 1. Four out of 18 CK-positive patients with early breast cancer had HER2-positive primary tumors (22 %; Table 2). In ten out of these 18 patients (55.6 %), the HER2 status in the primary tumor was different from that of CTCs, with a concordance rate of 44.4 % (in eight out of 18 patients). HER2-positive disease was encountered in ten (35.4 %) out of the 23 CK-positive patients with metastatic disease, whereas the concordance with CTC status was 55 %.

In order to investigate the co-expression of full-length HER2 and p95HER2 receptor in primary tumor cells, further triple-staining experiments were performed. Six patients with HER2-positive primary tumors (three with early and three with metastatic disease) were evaluated for the expression of p95HER2 receptor in primary tumors and CTCs. In three of these patients (one early and two metastatic), p95HER2 expression could be identified in an area covering approximately 5–10 % of the primary tumor; unfortunately, no CTCs could be detected in these patients at the time of the screening. Conversely, in one patient with detectable p95HER2-positive CTCs, truncated HER2 expression was not identified in the respective primary tumor. Table 2 summarizes the results concerning the HER2 status in the primary tumor and CTCs.

### Expression of p95HER2 receptor before and after first-line trastuzumab-based chemotherapy in metastatic breast cancer patients with HER2-positive primary tumors

**Fig. 4:**
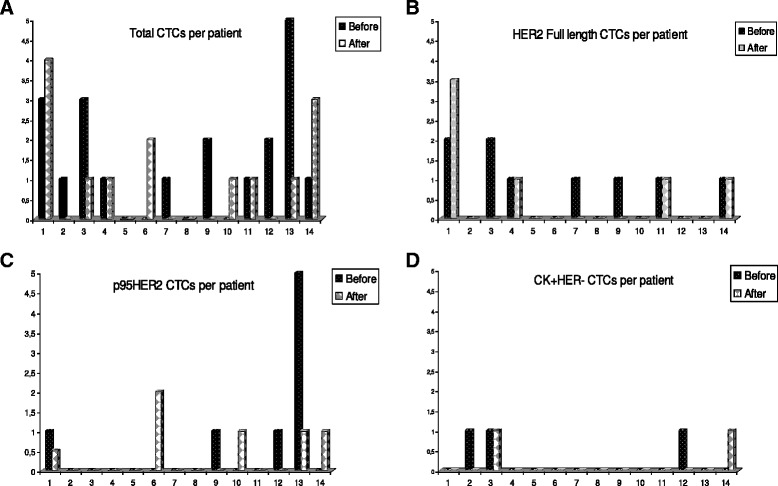
HER2 alterations on CTCs of metastatic breast cancer patients (*n* = 14) after trastuzumab administration. **a** Number of total CTCs per patient before and after trastuzumab-based chemotherapy. **b** Number of CK-positive/HER2-positive-expressing CTCs per patient before and after trastuzumab-based chemotherapy. **c** Number of p95HER2-expressing CTCs per patient before and after trastuzumab-based chemotherapy. **d** Number of CK-positive/HER2-negative-expressing CTCs per patient before and after trastuzumab-based chemotherapy.*CK* cytokeratin, *CTCs* circulating tumor cells, *HER2* human epidermal growth factor receptor 2, *p95HER2* the truncated form of HER2

Fourteen patients were screened for CK-positive cells before and after first-line trastuzumab-based chemotherapy. Ten of them had CK-positive cells before treatment; after treatment, the CTC numbers were reduced in six patients, remained unchanged in four and were increased in the remaining four patients (Fig. 4a).

Triple-staining experiments revealed that the full-length receptor was expressed in seven out of 14 patients before treatment and in four patients after trastuzumab administration (*p* = 0.035) (Fig. 4b). The p95HER2 receptor was expressed in four out of 14 patients before the initiation of therapy and in five after the completion of treatment (*p* = 0.45) (Fig. 4c). In addition, HER2-negative CTCs could be detected in three and four patients before and after trastuzumab administration, respectively (Fig. 4s).

The percentage of CTCs that expressed the full-length HER2 receptor was 58.3 % before treatment as compared to 40.1 % after treatment (*p* = 0.08). The population of p95HER2-positive CTCs numerically increased post-therapy (from 23.3 % at baseline to 43.23 % post-treatment) but this difference could not reach statistical significance (*p* = 0.59). HER2-negative CTCs were 18.3 % of the total examined CTCs before treatment and 16.7 % post-treatment (*p* = 0.7).

The median expression of p95HER2-positive CTCs per patient before and after treatment with trastuzumab was 0 % and 22.92 %, respectively. In addition, the median expression of the full-length HER2 receptor, on CTCs per patient, was decreased from 66.7 % to 16.67 % after treatment (*p* = 0.717). Before treatment, one out of 14 patients had exclusively p95HER2-positive CTCs while post-treatment three additional patients had CTCs exclusively expressing the truncated HER2 receptor.

### CTCs' phenotypic profile and clinical outcome

After a median follow-up period of 29 months (range, 0–74), 19 patients with metastatic disease had died. Furthermore, all patients with detectable truncated HER2-positive CTCs had died; Cox regression analysis revealed that OS of patients with detectable p95HER2-expressing CTCs was significantly decreased compared to patients without p95HER2-expressing CTCs (11.5 months versus 29 months; *p* = 0.03). In addition, patients with CK-positive/HER2-negative CTCs had a significantly decreased OS compared to patients with CK-positive/HER2-positive CTCs (5 months versus 42 months; *p* = 0.001) (see Figure S1 in Additional file 1). Furthermore, as shown in Table 2, p95HER2-positive CTCs could be detected in patients irrespectively of the hormone receptor status of the primary tumor. Moreover, there was no correlation between the HER2 expression on CTCs and the ER/PR status of the primary tumor (*p* = 0.539, *p* = 0.541).

Taking into account the status of the primary tumor, HER2-negative patients with CK-positive/HER2-negative CTCs experienced a significantly decreased OS compared to patients with CK-positive/HER2-positive CTCs (17 months versus 42 months; *p* = 0.018). Interestingly, although the number of patients with HER2-positive primary tumors is small, the detection of CK-positive/p95HER2-positive CTCs (*n* = 3) was associated with a significantly decreased OS compared to patients (*n* = 6) with CK-positive/p95HER2-negative CTCs (41 months versus 51 months; *p* = 0.021).

Statistical analysis in early breast cancer patients revealed no significant differences.

## Discussion

The presence of CTCs before and after the completion of adjuvant chemotherapy is associated with poor clinical outcome [22–24]. The same observation applies to metastatic patients [25, 26]. Although CTCs' phenotypic characteristics are not always the same as the primary tumor, the detection and characterization of CTCs could theoretically provide novel therapeutic targets for the prevention of metastatic process [13].

It is well known that HER2 is expressed in CTCs of early and metastatic breast cancer patients. The incidence of HER2 expression is usually higher in CTCs compared to the corresponding primary tumors [13–15]. HER2-targeted therapies have changed the outcome of patients with HER2-positive primary tumors [6]. We hypothesize that the expression of HER2 in CTCs could also be of clinical relevance. Recently, we reported that the administration of trastuzumab, in patients with HER2-negative early breast cancer who had detectable HER2-positive CTCs after the completion of adjuvant chemotherapy, resulted in a significant reduction of clinical relapses and a longer disease-free survival compared to patients who received only adjuvant chemotherapy [19]. Other studies have also reported similar results [27].

In the current study, HER2 receptor-expressing CTCs were detected in 55.6 % and 65.1 % of early and metastatic breast cancer patients, respectively. These results are compatible with previous studies using different methods for HER2 detection [13–15] and further support the already described discordance of HER2 status between the primary tumors and the corresponding CTCs, both in patients with early and metastatic disease [13, 28]. Furthermore, the observed discrepancy in the current study could be more likely attributed to the disease evolution and/or to tumor heterogeneity rather than the immunocytochemical HER2 detection in the primary tumor, which was based on a well-established standard methodology. Indeed, the Herceptest that was used in the current study contains an antibody that recognizes the intracellular domain of the protein, and thus, it can bind both the p95HER2 and the full-length HER2 receptor.

Recent studies have shown that not only the full-length HER2 but also the p95HER2 truncated receptor is crucial for the outcome of patients with breast cancer. p95HER2 lacks the extracellular domain of the receptor and is considered to confer a more aggressive phenotype [3, 5]. Particularly, p95HER2 expression in the primary tumor is associated with lymph node metastasis and reduced 5-year disease-free survival [4, 5] and emerged as an independent prognostic factor associated with a poor clinical outcome [2]. Moreover, in patients with metastatic breast cancer increased expression of p95HER2 is associated with a significantly decreased OS and PFS compared to patients with low p95HER2 expression [29].

To the best of our knowledge, there are no data in the literature concerning the p95HER2 expression in CTCs. In the current study, a new triple immunofluorescence assay was developed for the differential staining of the HER2 extracellular/intracellular domains in combination with the pancytokeratin antibody. A limitation of this assay is the use of one pancytokeratin antibody (A45-B/B3) instead of a cocktail [30] due to technical reasons. However, A45-B/B3 is the most commonly used antibody in the literature for the identification of CTCs [14, 15, 31–33]. Our results clearly demonstrate the presence of CTCs expressing the truncated HER2 form of the receptor in patients with early (11.1 %) but, more frequently, with metastatic (39.1 %) disease (*p* = 0.047). This observation is difficult to explain and suggests that CK-positive/p95HER2-positive CTCs may prevail during disease progression. It is also interesting to note that CTCs expressing exclusively the p95HER2 but not the full-length HER2 receptor were observed only in patients with metastatic disease. On the other hand, triple-staining of the primary tumors of six HER2-positive patients, revealed that, in three of them, p95HER2 could be identified in 5–10 % of tumor cells as already has been described [10]. Unfortunately, these patients had no detectable CTCs in their blood and, therefore, no correlations between the presence of p95HER2 in the primary tumor cells and CTCs were possible. In addition, another patient had p95HER2-positive CTCs but her primary tumor was negative for the truncated receptor. This observation suggests that the acquisition of p95HER2 on CTCs could represent a dynamic biomarker associated with molecular changes of tumor cells during disease evolution.

The expression of p95HER2 receptor in patients with HER2-positive primary tumors has been associated with resistance to trastuzumab; indeed, Scaltriti et al. [10] reported that patients with p95HER2 expression in their primary tumors failed to respond to trastuzumab-based regimens. Furthermore, Sperinde et al. [34] reported that metastatic breast cancer patients, with high expression of p95HER2 on the primary tumor, experienced a shorter progression-free and overall survival when treated with trastuzumab-based regimens. Our data concerning the effect of front-line trastuzumab-based chemotherapy on the expression of HER2 receptor in CTCs showed that treatment resulted in a statistically significant decrease (*p* = 0.035) of the CK-positive/HER2-positive CTC subpopulation. This finding is in accordance with previous findings that trastuzumab-based chemotherapy could effectively eliminate CK-positive/HER2-positive CTCs [35]. Conversely, CK-positive/p95HER2-positive CTCs were increased after treatment, a finding which could be attributed to a treatment-induced “selection” of the CK-positive/p95HER2-positive CTCs, since trastuzumab is known to bind to the extracellular domain of the HER2 receptor and, thus, p95HER2-expressing CTCs, lacking the extracellular domain of the receptor, may “escape” trastuzumab treatment.

Finally, preliminary analysis of overall survival in metastatic breast cancer patients revealed that the presence of CTCs expressing the p95HER2 phenotype is associated with a poor clinical outcome in terms of OS suggesting that the expression of the truncated form of HER2 receptor not only in the primary tumor [2, 34] but also in CTCs seems to be a poor prognosticator. These clinical correlations should be confirmed in future studies in larger groups of patients. In addition, the longitudinal evaluation of the HER2 phenotype of CTCs during the evolution of disease could provide a noninvasive biomarker which could be exploited for individually tailoring patients’ treatment.

## Conclusions

The presented results suggest that the p95HER2 receptor is expressed in CTCs of patients with both early and metastatic breast cancer, though the incidence is higher among metastatic patients, suggesting a possible relationship with the stage of disease. Furthermore, the prevalence of CK-positive/p95HER2-positive CTCs was increased after the administration of trastuzumab-based chemotherapy, implying that treatment could lead to the selection of HER2 subclones. It could be of interest to investigate whether dual inhibition of HER2 with trastuzumab and lapatinib is associated with a more effective eradication of both HER2-positive and p95HER2-positive CTCs, in order to provide an explanation for the increased clinical efficacy of this combination in HER2-positive breast cancer patients.

## Additional file

Additional file 1: Figure S1. Reduced OS in patients harvesting p95HER2-positive and CK-positive/HER2-negative CTCs. (TIFF 40 kb)

## Abbreviations

CK: cytokeratin
CTCs: circulating tumor cells
DFI: disease-free interval
DFS: disease-free survival
ECD: extracellular domain
EGFR: epidermal growth factor receptor
ER: estrogen receptor
FBS: fetal bovine serum
HER2: human epidermal growth factor receptor 2
ICD: intracellular domain
IgG: immunoglobulin G
OS: overall survival
p95HER2: the truncated form of HER2
PBMCs: peripheral blood mononuclear cells
PBS: phosphate-buffered saline
PFS: progression-free survival
PR: progesterone receptor
RT-PCR: reverse transcription polymerase chain reaction
