# Supplementary figures and images for: Expression of truncated human epidermal growth factor receptor 2 on circulating tumor cells of breast cancer patients
Source: Breast Cancer Res. 2015 Aug 19;17(1):113. doi: 10.1186/s13058-015-0624-x (PMC4541732; doi:10.1186/s13058-015-0624-x)

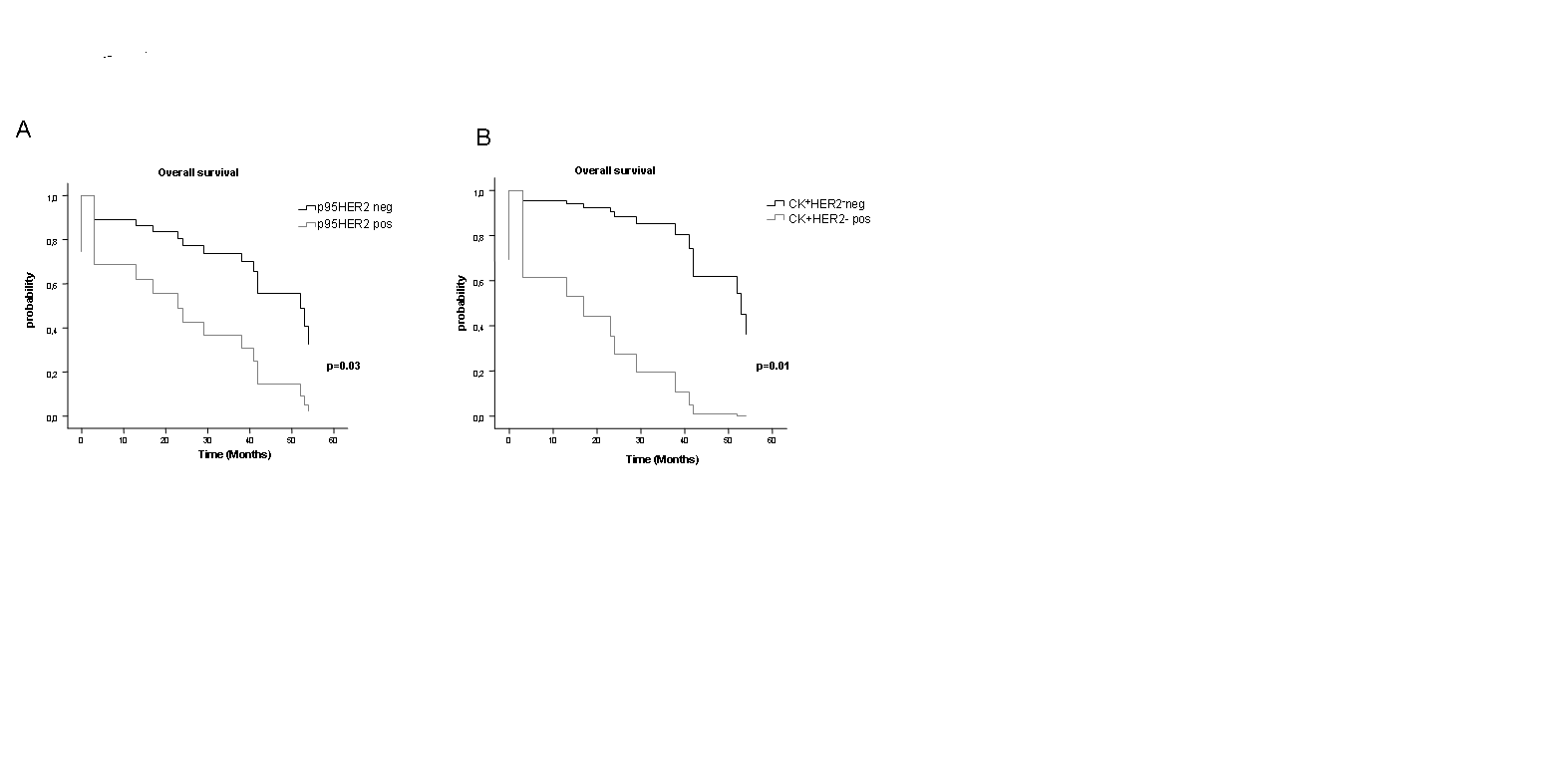

Supplement: Additional file 1: Figure S1. — Reduced OS in patients harvesting p95HER2-positive and CK-positive/HER2-negative CTCs. (TIFF 40 kb) [file 13058_2015_624_MOESM1_ESM.tif]
